# Supplementary material for: Co-Expression Network Analysis and Hub Gene Selection for High-Quality Fiber in Upland Cotton (Gossypium hirsutum) Using RNA Sequencing Analysis
Source: Genes (Basel). 2019 Feb 6;10(2):119. doi: 10.3390/genes10020119 (PMC6410125; doi:10.3390/genes10020119)
Supplement: Supplementary file 1 [file genes-10-00119-s001.zip › Supplementary Files/Abbreviations list.docx]

DPA days post anthesis

DEG differentially expressed gene

RILs recombinant inbred lines

RNA Ribonucleic Acid

QTL quantitative trait loci

GO gene ontologyWGCNA weighted gene co-expression network analysis

STEM Short Time-series Expression Miner

FPKM Fragments per kilobase of exon per million reads mapped

KEGG Kyoto Encyclopaedia of Genes and Genomes
