# Supplementary figures and images for: Co-Expression Network Analysis and Hub Gene Selection for High-Quality Fiber in Upland Cotton (Gossypium hirsutum) Using RNA Sequencing Analysis
Source: Genes (Basel). 2019 Feb 6;10(2):119. doi: 10.3390/genes10020119 (PMC6410125; doi:10.3390/genes10020119)

a

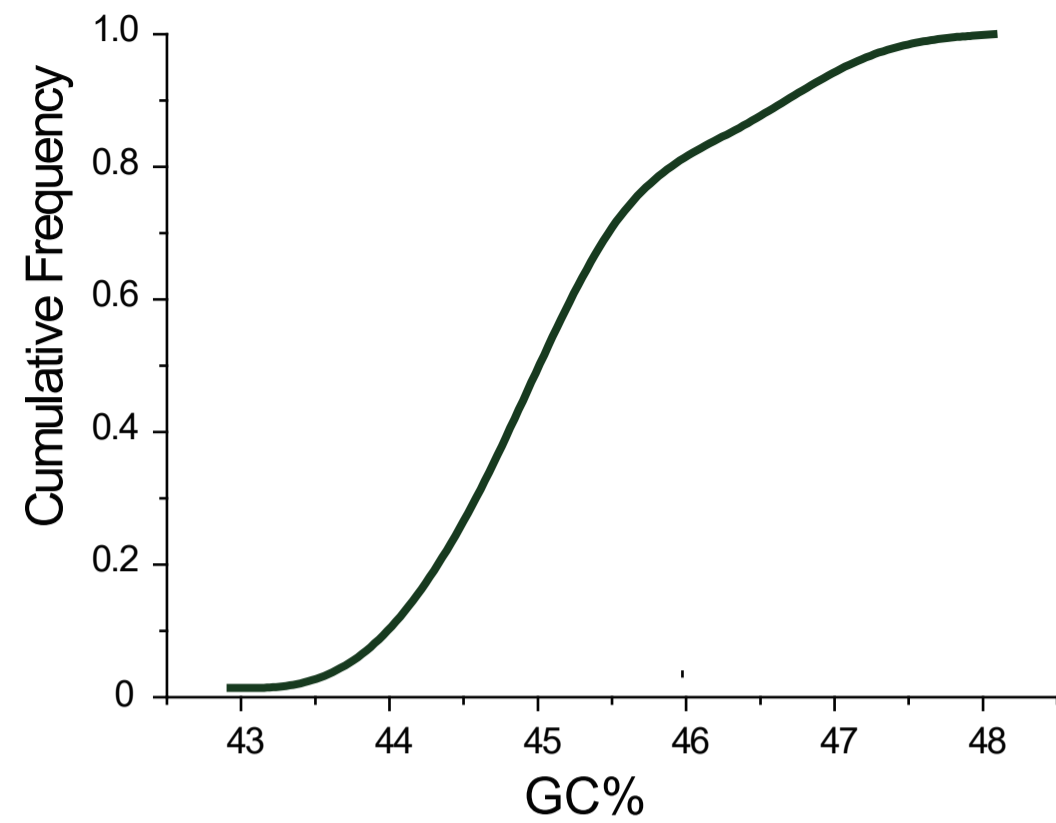

b

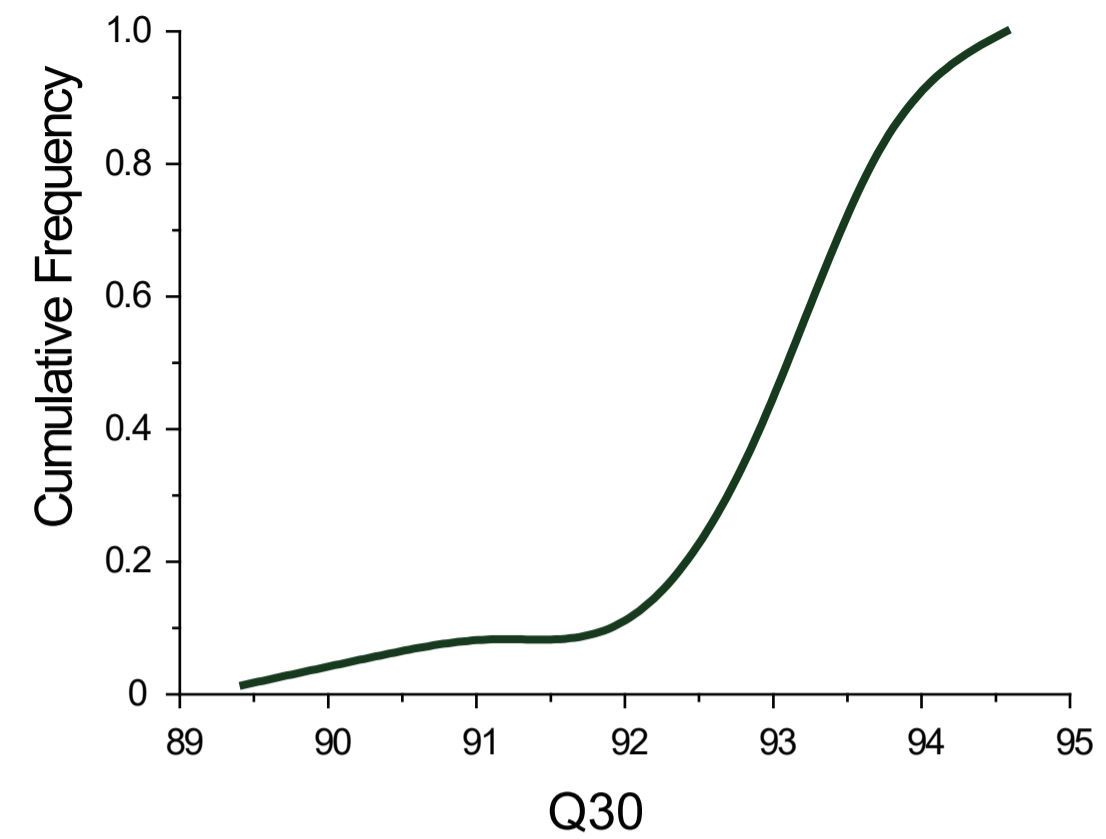

c

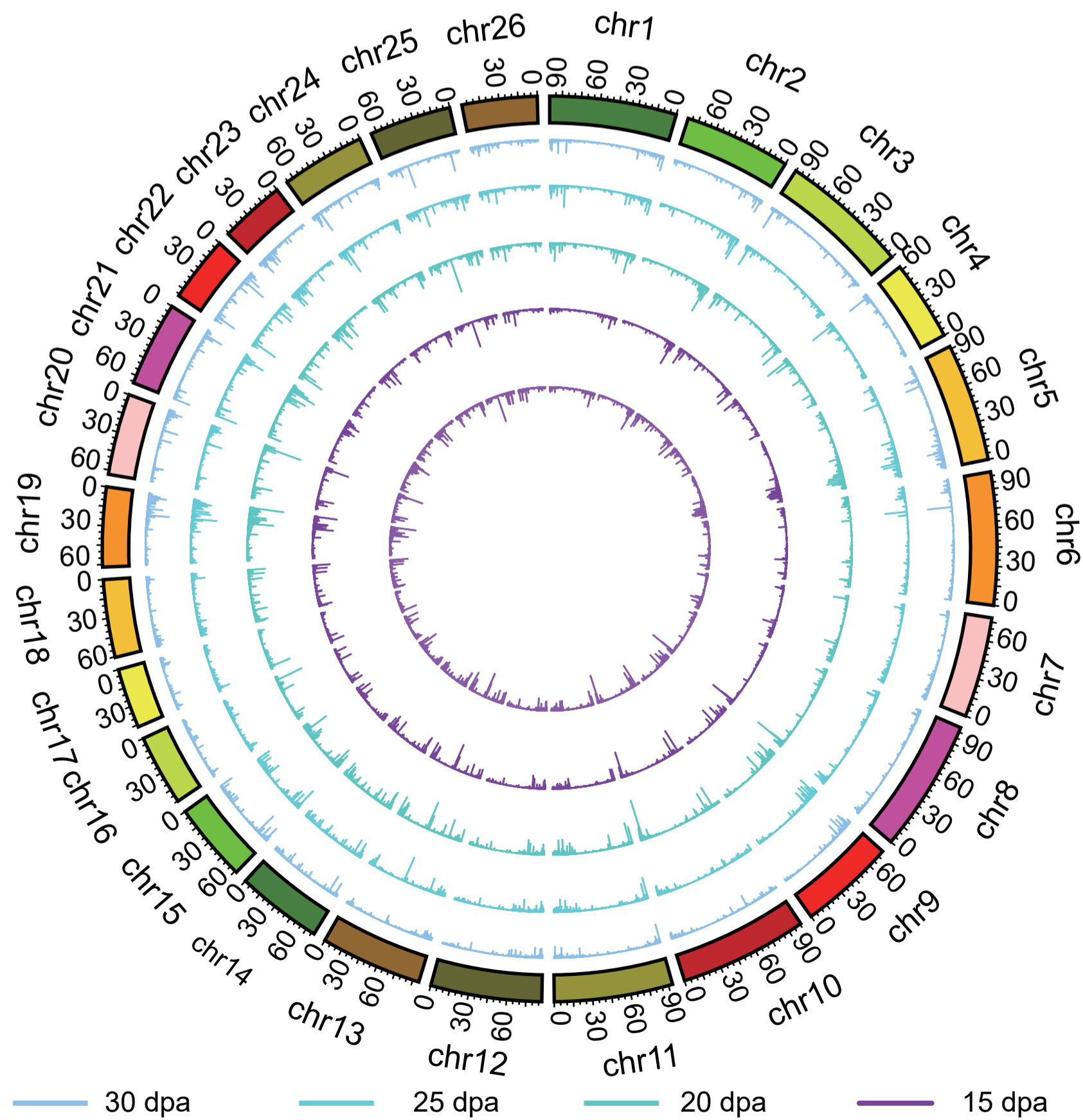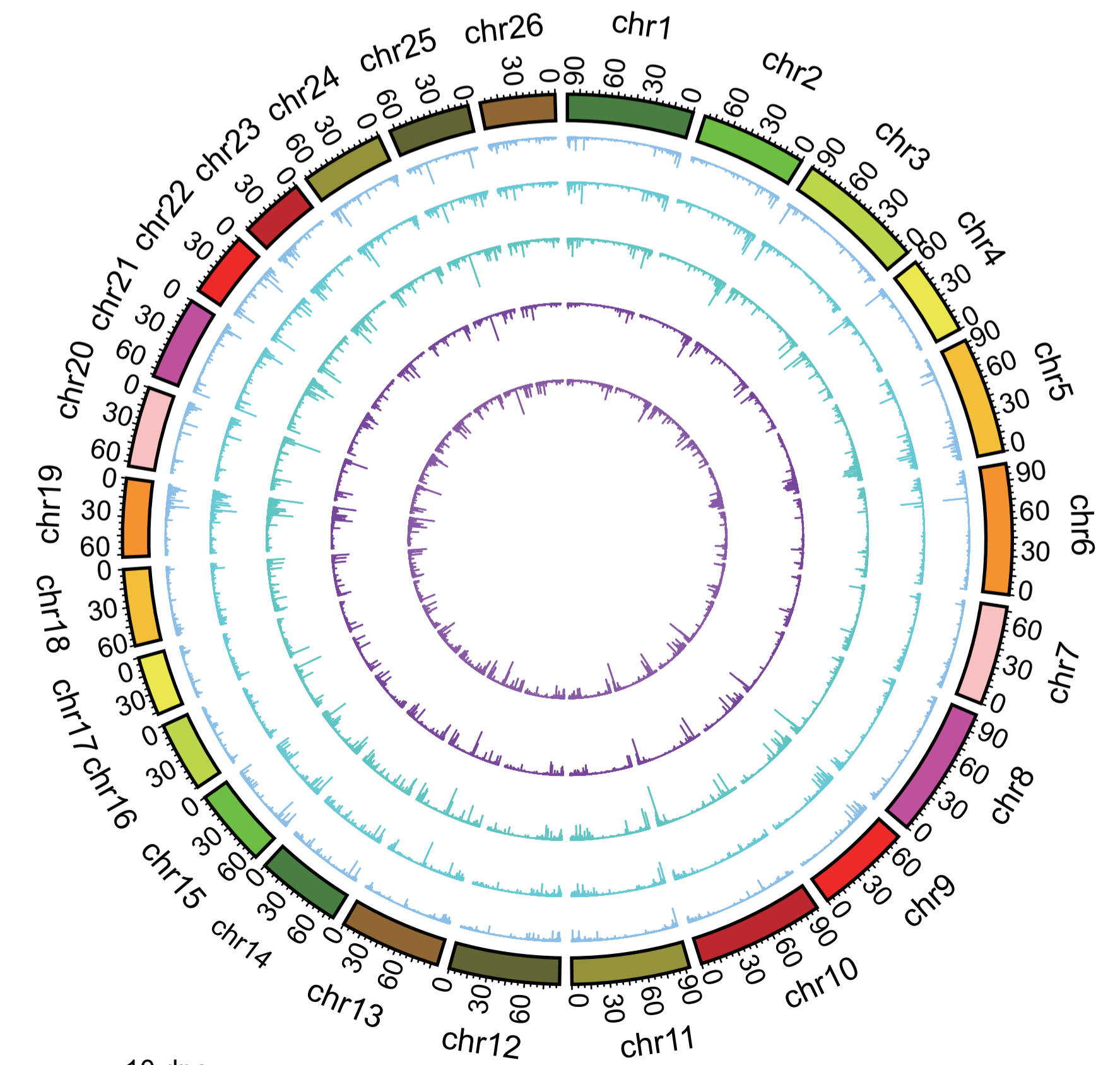

Supplement: Supplementary file 1 [file genes-10-00119-s001.zip › Supplementary Files/Figure S1.pdf]

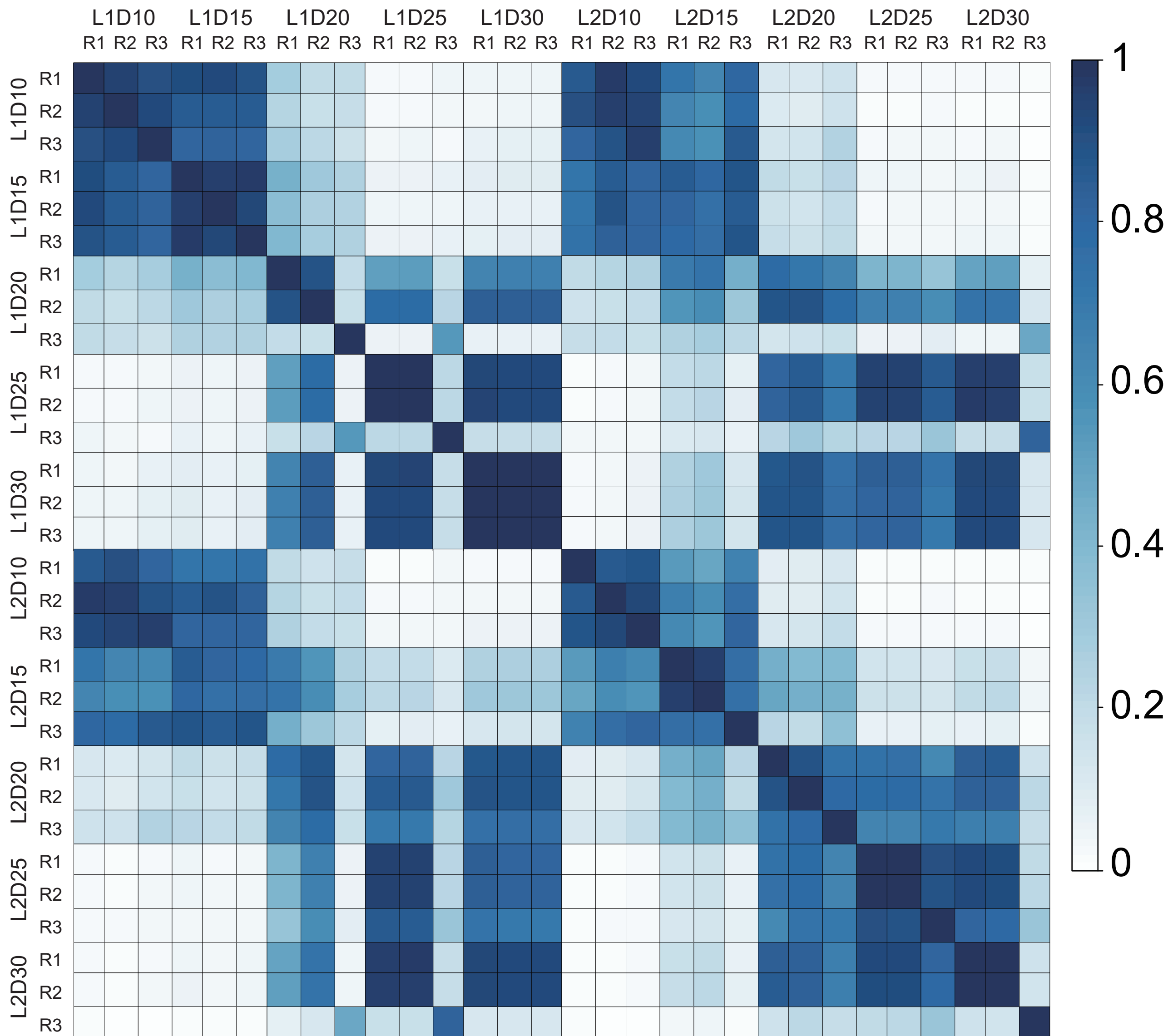

Supplement: Supplementary file 1 [file genes-10-00119-s001.zip › Supplementary Files/Figure S2.pdf]

a

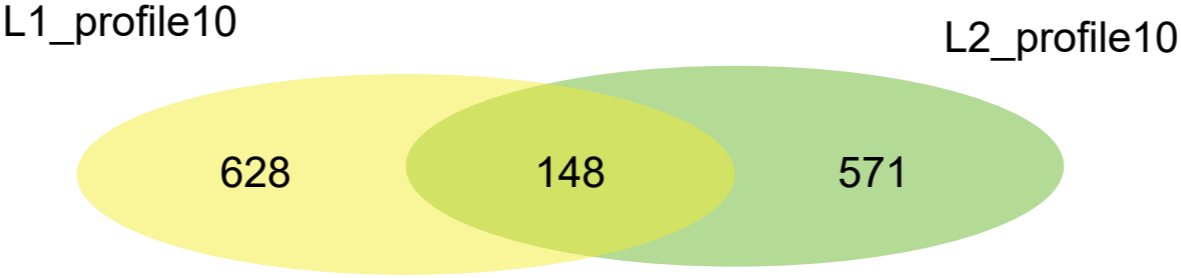

b

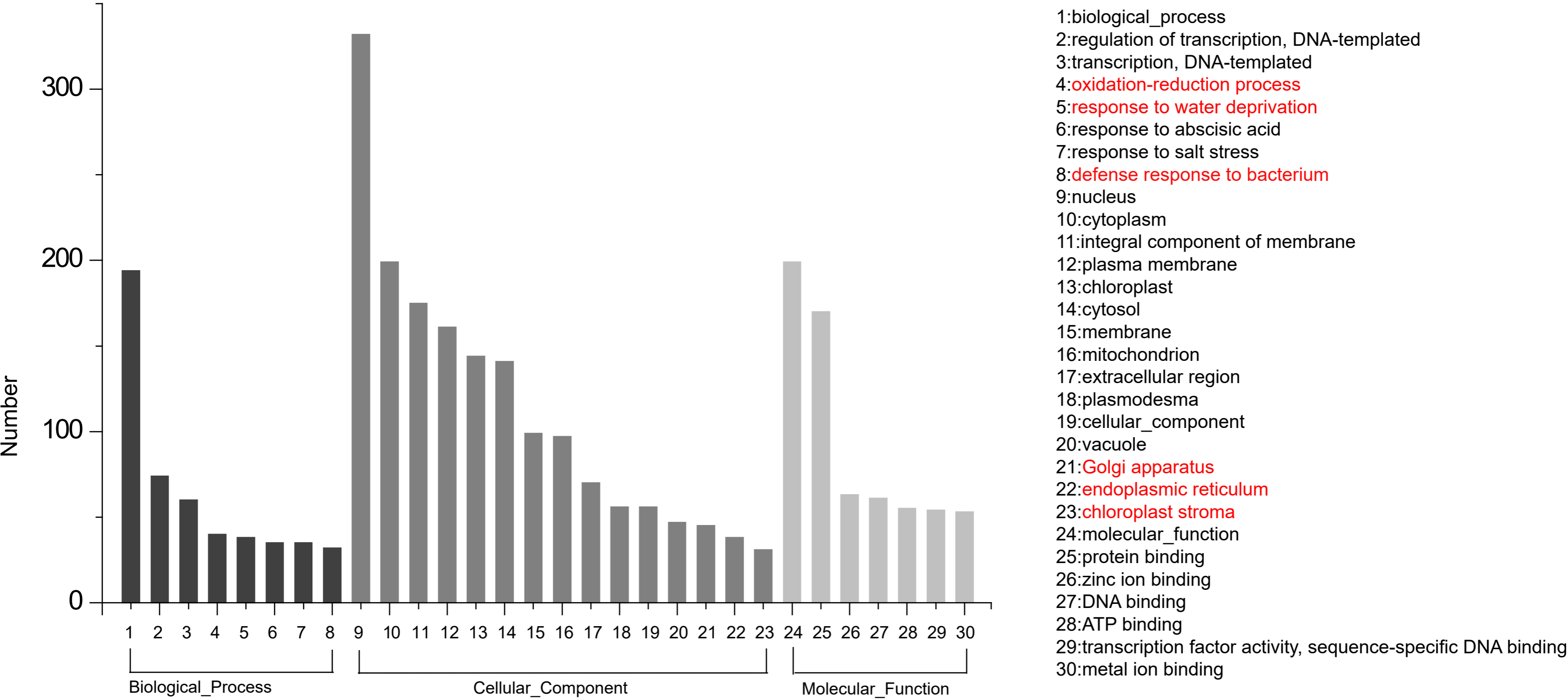

c

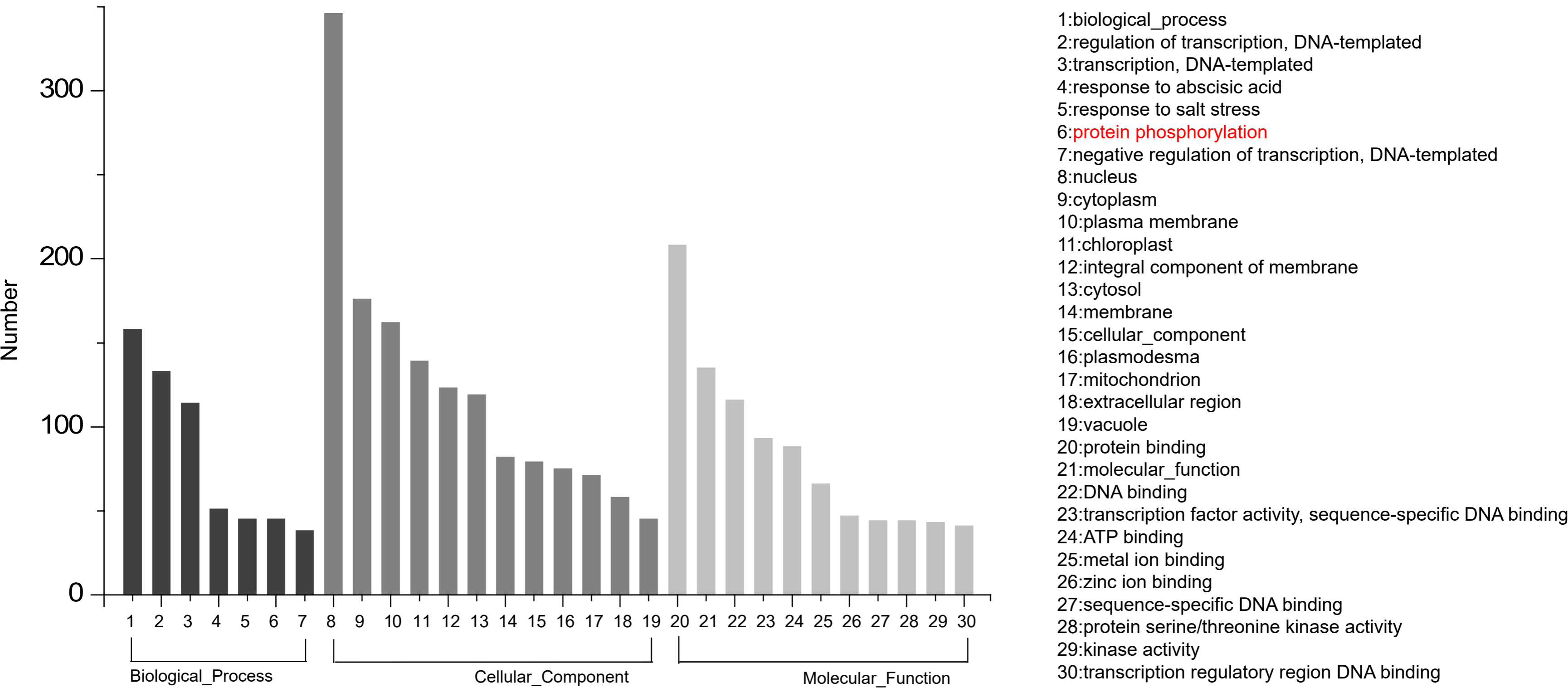

Supplement: Supplementary file 1 [file genes-10-00119-s001.zip › Supplementary Files/Figure S3.pdf]

a

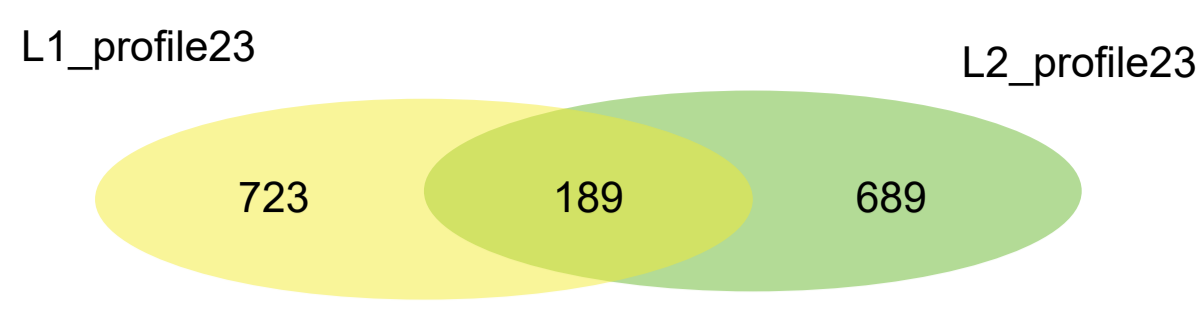

b

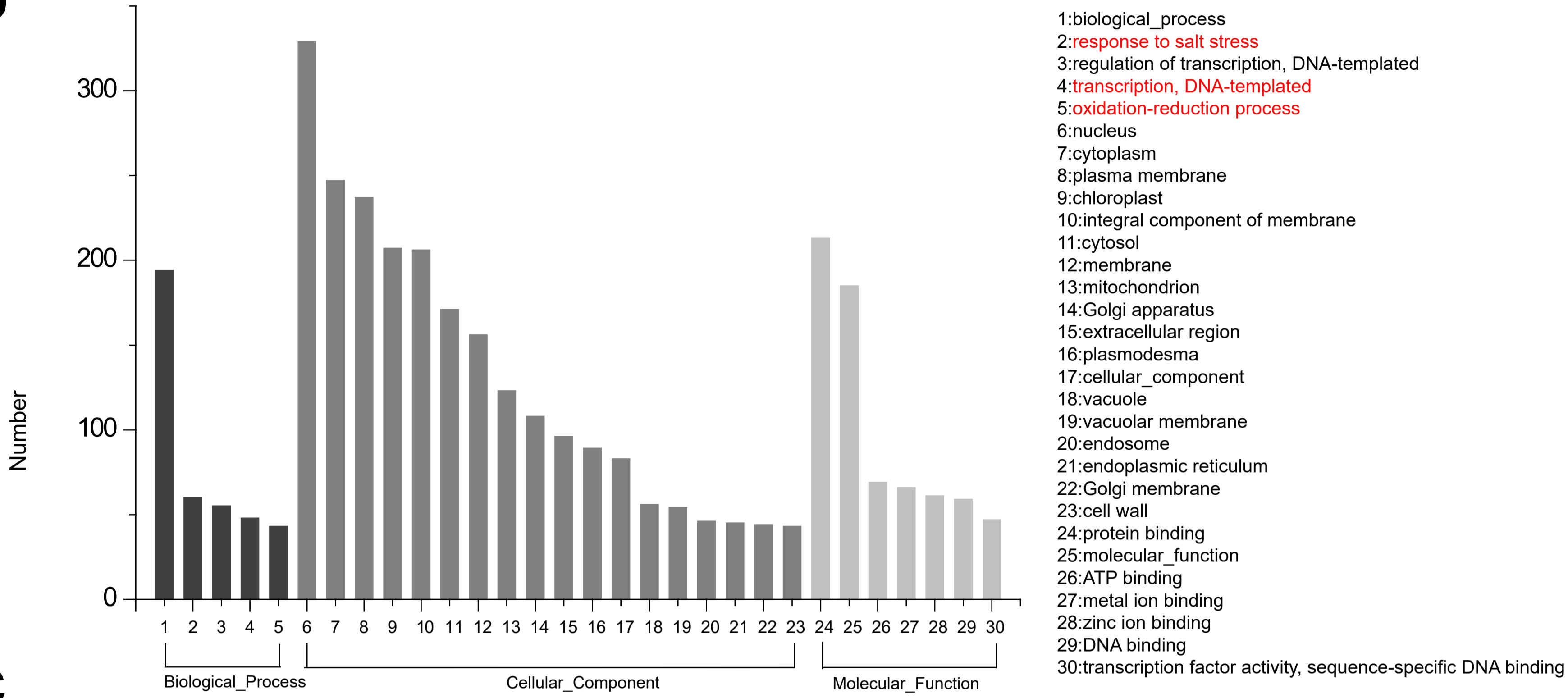

c

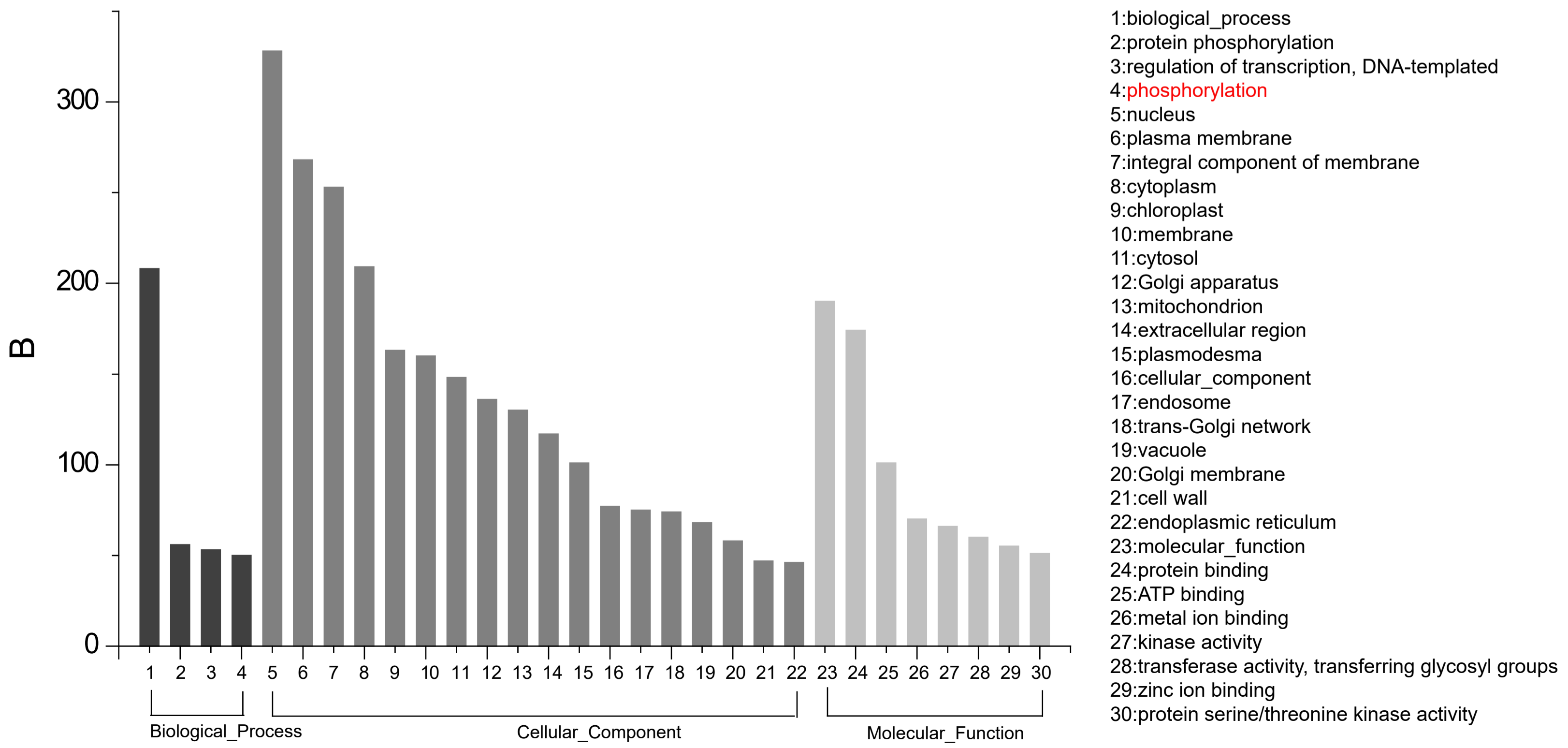

Supplement: Supplementary file 1 [file genes-10-00119-s001.zip › Supplementary Files/Figure S4.pdf]
